# An Impasse at the Royal Victoria Infirmary, Newcastle

**Published:** 1908-03-07

**Authors:** 


### HOSPITAL ADMINISTRATION.

### CONSTRUCTION AND ECONOMICS.

### AN IMPASSE AT THE ROYAL VICTORIA INFIRMARY, NEWCASTLE.

THE HOSPITAL CHAPEL.

At the annual meeting of the Governors of the Royal Victoria Infirmary, Newcastle, held on Saturday last under the presidency of the Lord Mayor of Newcastle, the question of the use of the infirmary chapel gave rise to an animated discussion. The chapel of St. Luke was consecrated by the late Bishop Lloyd for the use of members of the Church of England. Such consecration was with the consent and approval of the Governors of the Infirmary, and since then services have been held, in accordance with the rules prevailing at every hospital where the majority of patients belong to the Church of England. At other hospitals—for example, at the majority of our large metropolitan charitable institutions—the chapel is as much an adjunct of the hospital as are the wards. Its usefulness it would be difficult to over-estimate, and its worth to an institution which holds so many members of the Established Church, who are by their duties or through sickness debarred from attending services at neighbouring places of worship, is indubitable.

At Newcastle, however, the chapel has been a grievance. A large number of patients who enter the infirmary do not belong to the Established Church, but are members of one or other of the various evangelical sects who are classed, by the layman at least, under the generic title of Nonconformists. Such patients have conscientious scruples against attending the ordinary services according to the rites of the Established Church, which are held daily in the Infirmary Chapel for the benefit of inmates of the institution. Their objections were voiced, and voiced strongly and forcibly, at the annual meeting of the Governors. The committee of the hospital has, we understand, offered the Nonconformists the use of the students' lecture hall for occasional services—an offer which has been refused on grounds which have not been disclosed. At the meeting a resolution was tabled to the effect that the Chapel of St. Luke should be closed, and that no facilities should be offered to either party for the holding of religious services. An amendment to this resolution was proposed, requesting the committee to consider the advisability of offering to the Nonconformists a site within the precincts of the Infirmary for the erection of a chapel to be used by them for services. The original chapel was built out of funds set aside for that purpose from the general funds of the hospital, and the amendment proposed that the expenses of the new chapel should be defrayed by the Nonconformists, but that in view of the fact that the old chapel had been erected out of the general funds, members of the Church of England should be invited to contribute to the building fund. This amendment was put to the meeting, but it was apparent that the feeling of the whole meeting was against it. The amendment was defeated by 305 votes against 164, and the original resolution declared carried by a large majority.

The resolution of the Governors of the Newcastle Infirmary lays down a principle which is far reaching. It declares, in effect, the absolute undenominationalism of hospitals, and puts forward the view that the religious convictions of patients are matters of private concern that do not fall within the scope of hospital politics. To a very great extent that principle is a true one, but we cannot but consider that the amendment proposed at the meeting supplied a fair and equitable way out of a difficulty which from time to time must be faced by every charitable institution. In cases where a denomination is rich and influential enough to build and equip its own chapel it is only just that it should be given an opportunity to do so. The churches have been and are staunch supporters of hospitals, and their claims should have the sympathetic consideration of hospital committees.

We are without information as to whether the business of the chapel had been prominently brought before all the Governors previous to the meeting on the 29th ultimo. It would appear as if a whip had been sent round by one party, and the results of the voting may therefore be considered as probably not representing the opinion of anything like a majority of the Governors. We hope this may prove to be the case, as it is unthinkable that in this country any body of governors of a great general hospital should deliberately and finally determine to deprive the in-patients of a great hospital of all spiritual ministration and opportunities for worship during the period of their illness. Besides, the services in the chapel, as all administrators know, have formed the basis upon which has rested the *morale* of many an institution, and they have led to momentous results for good, not only directly to members of the staff and the patients, but indirectly by the influence they have exercised upon all who have had to do with the beneficent work of a great hospital. We cannot believe for one moment that the Governors of the Royal Victoria Infirmary, Newcastle, will ever permit the decision arrived at by a majority on Saturday to stand. If they do, not only must the Royal Victoria Infirmary suffer in reputation and *morale*, but it is likely to suffer materially in many ways, and it may even cease before long to be the great institution which it has been during so many years. We hope that on calm reflection all parties will unite to bring about an amicable adjustment, whereby the services and use of the chapel may be continued as heretofore, and an adequate provision may be made for the religious requirements of all denominations, the members of which from conscientious or other motives decline to use the present chapel. The sooner steps are taken in this direction and arrangements are made to summon a special meeting of the Governors to reconsider the whole question, the better it will be for the reputation of the Royal Victoria Infirmary, the welfare of its patients, and the credit of everybody connected with its administration.

